# Incidence, functional outcomes and cure rate of hematogenous infection in a 2,498 Total Knee Arthroplasties cohort

**DOI:** 10.1186/s40634-023-00656-2

**Published:** 2023-09-25

**Authors:** Daniel Pérez-Prieto, Albert Pardo, Albert Fontanellas, Joan Gómez-Junyent, Pedro Hinarejos, Joan-Carles Monllau

**Affiliations:** 1 Hospital del Mar – Universitat Pompeu Fabra (UPF), Passeig Marítim, 25, Barcelona, Spain; 2 IcatKnee, Hospital Universitari Dexeus – Universitat Autònoma de Barcelona, Barcelona, Spain

**Keywords:** PJI, TKA infection, Hematogenous PJI, DAIR, Late acute hematogenous infection

## Abstract

**Purpose:**

The primary aim of the present study is to report the late acute hematogenous (LAH) prosthetic joint infection (PJI) cure rate following Total knee arthroplasty (TKA) treated by means of debridement, antibiotics, and implant retention (DAIR) in a long-term follow-up. The secondary purpose is to report the functional outcomes at that follow-up and to compare them with a non-infected group.

**Material and Methods:**

This study cohort consists of 2,498 TKA performed from September 2005 to April 2010 that had a minimum follow-up of 10 years. The diagnosis of PJI and classification into LAH was done in accordance with the Zimmerli criteria. The primary outcome was the failure rate, defined as death before the end of antibiotic treatment, a further surgical intervention for treatment of infection, life-long antibiotic suppressive treatment or chronic infection. The Knee Society Score (KSS) was used to evaluate clinical outcomes.

**Results:**

Ten patients were diagnosed with acute hematogenous PJI during the study period (0.4%). All of them were managed with DAIR, which was performed by a knee surgeon and/or PJI surgeon. The failure rate was 0% at the 8.5-year (SD, 2.4) follow-up mark. The KSS score was 82.1 vs. 84.1 (p n.s.) at final follow-up.

**Conclusion:**

Although the literature suggests that TKA DAIR for LAH periprosthetic joint infection is associated with high rates of failure, the results presented here suggest a high cure rate with good functional outcomes.

**Level of evidence:**

Level II, prospective cohort study.

## Introduction

Periprosthetic Joint Infection (PJI) is a complication that has seen rising interest over recent decades with the improvement in its diagnosis, classification, and treatment. While the Zimmerli classification into acute postoperative, late acute hematogenous (LAH) and chronic PJI is still widely used, the diagnostic criteria have evolved since then with the recent European Bone and Joint Infection Society (EBJIS) criteria that is supported by societies worldwide [12, 13, 27]. In the case of surgical treatment, a lot has changed since Insall’s recommendation of a 2-stage approach for all PJI. In the early 2000s, the Debridement, Antibiotics and Implant Retention (DAIR) approach for both acute postoperative and late acute PJI was proposed [28]. The rationale was that most of the bacteria were in planktonic form and the biofilm formed in the prosthesis was “young” enough to be eradicated in the first weeks with antibiofilm antibiotics Additionally, it could be done without removing the part of the prosthesis that were attached to the bone. This method has been proven to be effective in several studies for acute postoperative PJI with a cure rate of 90% or superior [5, 6, 25]. Moreover, this strategy has obtained good functional outcomes with there being better function than with the one-stage or the two-stage prosthetic revision approaches [6, 7, 21].

However, different results have been published with some reports of a cure rate as low as 70% in cases of LAH PJI. That is one reason some authors advocate for other surgical approaches in LAH PJI [22–24]. In the case of Total knee arthroplasty (TKA), some studies report even a higher failure rate [10, 26].

The primary purpose of the present study is to report the LAH PJI cure rate following TKA treated by means of DAIR in a long-term follow-up. The secondary purpose is to report functional outcomes at that follow-up and to compare them with those of the non-infected group. The hypothesis is that DAIR provides a high cure rate in LAH PJI as well as functional outcomes like those of non-infected cases.

## Methods

This is an ambispective study. The initial clinical trial was approved by the Institutional Ethics Committee and the trial was registered with ClinicalTrials.gov (number NCT01631968). This study ended but the patient cohort was followed-up every 2 years and included in the same database. The patients agreed to participate by signing an informed consent document. A total of 3000 primary TKA in the period from September 2005 to April 2010 were included.

In the present study, this 2,498 TKA cohort had a minimum follow-up of 10 years. The remaining 502 were lost to follow-up.

The diagnosis of PJI and classification into LAH was done in accordance with the Zimmerli criteria [27]. The primary outcome was the failure rate, defined as death before the end of antibiotic treatment, a further surgical intervention for the treatment of infection, life-long antibiotic suppressive treatment or chronic infection. The Knee Society Score (KSS) was used to evaluate clinical outcomes at the last follow-up visit. The antibiotic treatment, the source of infection (primary focus) and the microorganisms isolated were also assessed. Whether the surgeon who performed the DAIR was a knee and/or PJI surgeon was also evaluated.

All the patients had primary TKA in the same surgical suite with laminar airflow exchange. No body exhaust suits were used. Patients with any diagnosis leading to TKA were included.

The only exclusion criteria were a history of infection in the knee or a history of allergy to one or both of the antibiotics used in the cement compound.

Preoperative intravenous prophylactic antibiotics were administered with 2 g of cefazolin to all patients in a 10 to 15-min infusion some 30 to 60 min before incision or 1 g of vancomycin was given in a 1-h infusion some 60 to 90 min before incision if the patient had a beta-lactam allergy. The antibiotic prophylaxis was complemented by 1 g of cefazolin every eight hours or 1 g of vancomycin every twelve hours for the first twenty-four hours after surgery.

DAIR was considered the treatment of choice for every LAH PJI and this approach was taken in every case if the Zimmerli criteria had been fulfilled. In this study, DAIR was performed as previously described [8]. It included arthrotomy, extensive synovectomy and thorough debridement. Irrigation was carried out by means of pulsatile lavage at the surgeon’s criterion (no less than 3 L). Exchange of the polyethylene was always performed, and the posterior compartment was debrided.

As part of the standard protocol for all prosthetic revisions, synovial fluid was aspirated and sent to determine the leukocyte count along with its differential count and for culture (in an EDTA tube). Five periprosthetic tissue specimens were collected from distinct surgical sites. The tissue specimens were crushed, and 0.5 mL of homogenate was plated on agar, and the remaining volume was inoculated in thioglycolate broth. All cultures were incubated at 37ºC for 7 days (aerobically) or 14 days (anaerobically) and inspected daily for microbial growth. The removed polyethylene was transported to the microbiology laboratory in air-tight containers. The prostheses were vortexed in containers for 30 s. Sonication was performed for 1 min at a frequency of 40 ± 5 kHz. The container was subsequently vortexed again for an additional 30 s and aliquots of 0.5 mL of sonication fluid were plated onto aerobic and anaerobic agar plates within 4 h after sonication and thioglycolate broth was inoculated with another 0.5 mL. The cultures were incubated at 37ºC for 7 days (aerobically) or 14 days (anaerobically) and inspected daily for microbial growth.

For the secondary aim of the study, a control group of 20 paired-matched patients (1:2) were selected from the same TKA cohort as can be seen in Table 2.

### Statistical analysis and sample size

Continuous and categorical variables are presented as means (with standard deviation, SD) and counts and percentages, respectively. Continuous data was compared between the groups with the Student's t-Test for independent samples and, over time, with the Paired Student’s t-Test. Categorical variables were compared with the Chi square or Fisher test, when appropriate. A bivariate analysis comparing each parameter with the assigned group was completed using the Chi Square or Fisher test as necessary. P values of < 0.05 were considered statistically significant. A propensity matching score was made to assess the covariates (antibiotic-loaded bone cement).

No power size calculation was needed for the main purpose as it was a descriptive one. For the second purpose, the Chi Square difference test was used to determine the sample size. Assuming a statistically significant difference of KSS greater than or equal to 5 units and a 10% rate of lost to follow-up, 9 subjects were necessary in the first group and 18 in the second group, establishing an α error of 0.05 and a statistical power of 80%.

The statistical analysis was done using SPSS 18.0 software package (SPSS Inc., Chicago, IL).

## Results

Among the 2,498 TKA procedures, 10 patients were diagnosed with acute hematogenous PJI during the study period (0.4%). Those 10 patients were managed with DAIR, which included the polyethylene exchange. They were performed by a knee surgeon and/or PJI surgeon. The failure rate was 0% at the 8.5-year (SD, 2.4) follow-up mark.

The elapsed time between primary total knee replacement surgery and the DAIR intervention was 4.7 years (SD, 3.6). DAIR was performed at 2.75 days (SD 1.8) of the onset of symptoms. The most common infecting organisms were *Staphylococcus aureus* (30%) and *Escherichia coli* (30%). There were 2 infections caused by coagulase-negative staphylococci and 2 culture-negative PJI. All culture-positive PJI microorganisms were susceptible to anti-biofilm antibiotics.

Six infections occurred in TKA cemented with antibiotic-loaded bone cement and 4 infections occurred in TKA cemented with plain cement (n.s.).

The primary focus of infection was identified in only 3 cases. The mean duration of antibiotic treatment was 11.4 weeks (SD 1.9).

The postoperative clinical outcomes were excellent, with a mean KSS of 84.1 points (SD, 14.6). More results can be seen in Table 1.

**Table 1:** Detailed results of the LAH TKA infection

Patient	Age (years)	Gender	Elapsed time (months)	Days to DAIR	Pathogen	KSS	Follow-up (years)
1	82	Female	4.46	4	*S. aureus*	96	8
2	78	Male	41.7	3	*S. epidermidis*	88	5
3	60	Female	58.1	29	*S. warneri*	66	6
4	72	Female	3.7	2	*S. aureus*	90	7
5	80	Female	45.3	6	*E. coli*	92	7
6	80	Female	4.2	4	*S. aureus*	67	8
7	73	Female	101	2	*E. coli*	47	9
8	64	Male	105.8	1	*-*	100	12
9	56	Female	109.9	1	*-*	95	12
10	81	Female	92.8	1	*E. coli*	100	11

As for the comparison between infected and non-infected cases, the outcomes were similar in all the studied items (p n.s.). Details of this assessment can be seen in Table 2

**Table 2:** Outcomes compared between infected and non-infected cases

	Control	Late acute hematogenous	Total	
	(*N* = 20)	(*N* = 10)	(*N* = 30)	*p*-value
**Age (years)**				0.441
Mean (SD)	70.70 (9.76)	72.60 (9.47)	71.33 (9.54)	
Median (Q1, Q3)	70.0 (67.0, 73.5)	75.5 (64.0, 80.0)	71.0 (67.0, 80.0)	
Min., Max	51.0, 91.0	56.0, 82.0	51.0, 91.0	
**KSS**				0.291
Mean (SD)	82.10 (10.70)	84.10 (17.87)	82.77 (13.23)	
Median (Q1, Q3)	84.0 (76.0, 91.0)	91.0 (67.0, 96.0)	85.0 (75.0, 92.0)	
Min., Max	57.0, 100.0	47.0, 100.0	47.0, 100.0	
**Follow-up (years)**				0.741
Mean (SD)	8.95 (2.96)	8.50 (2.46)	8.80 (2.77)	
Median (Q1, Q3)	8.5 (6.5, 11.0)	8.0 (7.0, 11.0)	8.0 (7.0, 11.0)	
Min., Max	4.0, 15.0	5.0, 12.0	4.0, 15.0	
**Gender**				1.000
Male	4 (20.0%)	2 (20.0%)	6 (20.0%)	
Female	16 (80.0%)	8 (80.0%)	24 (80.0%)	

## Discussion

The most important finding of this study in the present cohort is that LAH PJI was successfully treated by means of DAIR with a 100% cure rate at the 8-year follow-up. Moreover, the functional outcomes were similar to those of non-infected cases. In that sense, the hypotheses have been confirmed.

To the best of our knowledge, this is the first study following a TKA cohort to evaluate LAH PJI for such a long period. This study provides insight into the risk of having an incidence of LHA PJI during the first 8 years after implantation, which is 0.4%.

Although DAIR has been widely used in cases of both acute postoperative PJI and LAH PJI, several studies have reported a worse cure rate in cases of LAH in the recent years [17, 22–24]. The reasons the authors give for this higher failure rate in late acute staphylococcal infections might be an unrecognized chronic PJI that deteriorated acutely (with or without a secondary bacteremia) and that the infection was thus misclassified as an acute one [22]. However, in the present study, 50% of the infections were caused by staphylococcal species for which there was a high cure rate.

Contrary to the previously cited studies, different results have been obtained in the present study. One reason for this disparity may be the fact that all the DAIR were performed by a knee surgeon and/or PJI surgeon. This is very important as Borens et al. conclude that the PJI cure rate is higher when the surgery is carried out by a PJI surgeon. They usually perform a more aggressive and thorough debridement [8].

Another fact that may explain the result of the present study is that some literature may misdiagnose LAH PJI [9, 14]. To distinguish between LAH PJI and acute on chronic PJI, it may be difficult as the latter usually presents with mild to moderate pain for a long period of months or years (with no other symptom). Later, redness, swelling and worsening knee pain progressively develop over a shorter period of weeks or months. They seldom present with fever [11, 15]. On the contrary, patients suffering from LAH PJI typically have an uneventful course and no pain. Then, they suddenly (hours or maximum days) present with moderate to severe pain, a swollen knee and fever [8, 11, 15]. All 10 patients included in this study presented in the emergency room in that way.

Choi et al. found that not exchanging the polyethylene in DAIR is an independent risk factor for failure [4]. Moreover, Zhang et al. found similar results without impairment in the functional results [25]. In the present study, it is interesting to note that the functional results in LAH PJI treated by means of DAIR are like those in non-infected cases. Those results were also recently described by Barros et al. [2].

The source of infection (original foci) was only identified in 30% of the cases in the present study and none of them were related to invasive procedures. This is in agreement with the latest recommendations to avoid antibiotic prophylaxis in dental or endoscopic procedures [18, 20]. Moreover, the fact that 70% percent of the patients with an unknown foci LAH PJI have been cured may suggest that identification and a extensive work-up may not be needed except for very specific cases such as *Streptococcus bovis* or *Gemella morbillorum* and their relationship with intestinal tumors [3, 19].

Finally, Renz et al. have reported a lower cure rate of LHA streptococcal PJI [16, 17]. Some other studies have also concluded the same [1]. The reason may be that streptococcal biofilm is difficult to treat. This is important as the incidence of streptococcal infection in LAH PJI is around 20% [27]. However, none of the cases presented were due to this microorganism, which may be another reason for the high cure rate seen in this study.

There are some limitations to this study. The first one is that we have not reported all the LAH PJI procedures done at our center. Only the infections of the TKA cohort have been reported. At the same time, this is one strength of the study as it has made it possible to follow a large cohort of some 3.000 TKA for a long period and to determine the long-term risk of LAH PJI as well as the cure rate and the functional outcomes in a long-term follow-up. Another important limitation is the fact that host characteristics were not reported in this study. Finally, the resistance of the bacteria is also crucial for the results presented here. As previously stated, all the microorganisms were susceptible to antibiofim antibiotics. Therefore, these results may not be replicated if the resistance patterns are different.

## Conclusion

Although the literature suggests that TKA DAIR for LAH periprosthetic joint infection is associated with high rates of failure, the results presented here suggest a high cure rate with good functional outcomes.
